# The Genome Assembly Archive: A New Public Resource

**DOI:** 10.1371/journal.pbio.0020285

**Published:** 2004-09-14

**Authors:** Steven L Salzberg, Deanna Church, Michael DiCuccio, Eugene Yaschenko, James Ostell

## Abstract

With the genome assembly archive, it is possible to examine the raw data that underlies the DNA sequence in any sequenced genome

Scientists have dedicated considerable effort to decoding the genomes of an ever-growing list of species, ranging from small viruses, whose genomes may be just a few thousand nucleotides in length, to large mammalian genomes, three billion nucleotides and larger. Many aspects of life science research have benefited by the accumulation of these data, but decoded genomes could be even more valuable if important information about the genome sequence, currently being lost, were preserved. Occasionally, questions arise about a specific position in the sequence—or a variant in the sequence is observed in a new sample. At times like these, it would be helpful to be able to go back to the experimental evidence that underlies the genome sequence at that position, to see if there is any ambiguity or uncertainty about the sequence. As things stand, that's almost impossible. To understand why this is the case, it is necessary to know a bit more about how a genome sequence is put together.

Current sequencing technology can only generate 700–800 nucleotides at a time; genomes must therefore be shattered into many small fragments (in what is known as the “shotgun” approach), which are then sequenced. The sequences are assembled to generate a consensus sequence that, if all steps work perfectly, matches the original DNA molecule. Since the sequencing of Haemophilus influenzae in 1995 (Fleischmann et al. 1995), most bacterial and archaeal species have been sequenced by fragmenting the entire genome, sequencing the pieces, and assembling the result (the whole-genome shotgun, or WGS, strategy). In recent years, ever-larger sequencing projects have followed the WGS approach, requiring teams of computer experts and the use of increasingly sophisticated assembly algorithms in order to put together the huge number of sequence fragments.

Without really being aware of it, the bioinformaticians who assemble genomes have for years been discarding the valuable information on how all of the individual sequence fragments align to the assembled chromosomes. This loss has gone largely unremarked because the scientific community has focused its attention primarily on the end product: the final genome sequence itself. It is only natural to regard the genome sequence, which is the basis for gene discovery and for functional understanding of the biology of the organism, as the primary result of a WGS project. In reality, though, a WGS project is an experiment in which large numbers of sequencing reactions are run, followed by a combination of computational work and additional sequencing to complete the genome. Three years ago, the Trace Archive (at The National Center for Biotechnology Information and The Wellcome Trust Genome Campus in Hinxton, United Kingdom) was developed to store the raw sequence data and to facilitate dissemination of this data, but currently there is no database that captures the alignment of these reads to the published genome sequence.

Many scientists would be surprised to hear that genome assemblies are unavailable. One might infer that the assembly of a genome could be reconstructed from the genome sequence and the associated traces. However, aligning the traces to the genome will generally not reproduce the assembly, both because many of the traces will have alternate possible alignments and because, in some cases, parts of the assembly are manually refined based on additional experimental data. Furthermore, only a small number of large-scale centers have the computing hardware, software, and bioinformatics expertise to allow them to assemble a large genome.

To bridge this gap, we have developed the Assembly Archive (http://www.ncbi.nlm.nih.gov/projects/assembly). The archive has been developed to store both an archival record of how a particular assembly was constructed and the alignments of any set of traces to a reference genome. Assemblies contained in this archive will be available in the GenBank (http://www.ncbi.nlm.nih.gov/Genbank/index.html), DDBJ (http://www.ddbj.nig.ac.jp/), and EMBL (http://www.ebi.ac.uk/embl) databases, and all underlying traces are required to be deposited in the Trace Archive.

The Assembly Archive's first entries are a set of seven closely related strains of Bacillus anthracis (the causative agent of anthrax), which have been sequenced as part of an effort to understand the detailed variation of that species. This includes the completed reference genome of the Ames strain, sequenced from a sample kept frozen since 1981, when it was originally isolated in West Texas (J. Ravel, personal communication). For the first time, the evidence behind each polymorphism in these assembled genomes will be directly accessible to the scientific community.

## Microbial Forensics

Recently, heightened awareness of the threat of bioterrorism has spurred efforts to sequence genomes of multiple strains and isolates of a number of microbial pathogens, with the goal of cataloging all sequence differences between genomes. These efforts began with the study of the B. anthracis bacterium (the bacterium sent through the United States mail in late 2001) in order to determine if there were any differences between it and a reference laboratory sample (Read et al. 2002). This and subsequent studies have prompted many scientists to focus much greater attention on the assembly of a genome, and to regard the assembly rather than the genome as the object of greatest interest. In these forensic studies, we sequence whole genomes in order to discover every possible genetic difference between two bacteria or viruses. These genomes may differ in just one or two nucleotides out of millions that are identical; for example, the study referenced above uncovered just four single nucleotide polymorphisms (SNPs) in a chromosome of 5.23 million base pairs. The close similarity between the sequences forces us to consider all the facts behind each individual nucleotide that appears different. For studies that might be used as evidence in criminal investigations, it is essential to produce this information, and furthermore to quantify our confidence in each nucleotide in the genome. Regions of a genome with deep coverage are much more accurate than those with light coverage (i.e., regions with just one or two sequence reads).

**Figure 1:**
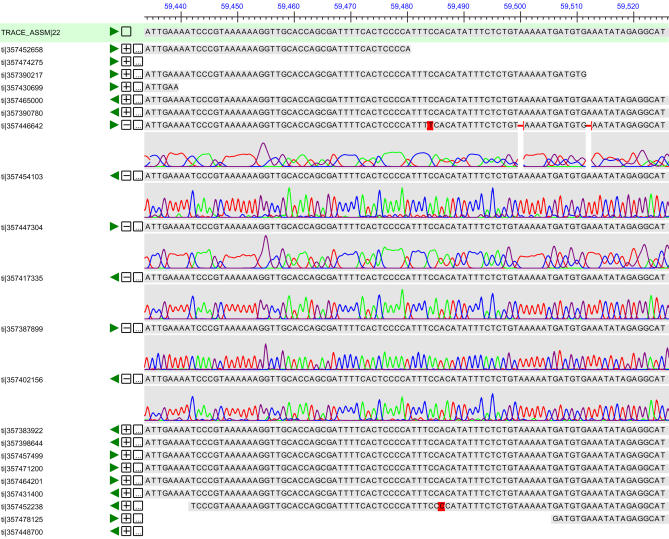
Snapshot of the Underlying Sequences and Traces from an Assembly of B. anthracis

Figure 1 shows one of the interfaces in the Assembly Archive, covering a small region of the multiple alignment of sequences and traces to one of the newly deposited anthrax genomes. It also shows how it is possible to examine the evidence underlying a specific base in the DNA sequence.

The consensus sequence shown across the top of the figure contains multiple sequences that validate each nucleotide in the window. Runs of a single base (monomer runs) are common causes of base-calling errors, because the peaks in the underlying trace data sometimes merge together. The sequence shown includes several monomer runs; several of the underlying traces are shown as well. For example, the run of six As at the far left of the figure is supported by several reads in which all six peaks are distinct, as well as other reads in which the six nucleotides appear as one broad peak. By examining data such as these, one can easily verify (or disprove) putative SNPs in this genome.

## Human SNP Research

Human polymorphism studies (e.g., Sachidanandam et al. 2001) are a tremendously active and important area of research today. SNPs are directly implicated in a large number of diseases and inherited traits (Risch 2000, Chakravarti 2001). Within “haplotypes,” they describe individual variation for drug response (McLeod and Evans 2001) and provide a genetic framework for understanding disease phenotype (Hoehe 2003).

In contrast with prokaryotic genomes, the human genome (as well as other animals, plants, and a broad range of eukaryotes) is diploid, and as a result many SNPs can be discovered within a single assembly, which contains the chromosomes representing the two parent organisms. SNPs can also be found through population studies in which the same locus is sampled from multiple individuals. In either case, the evidence for a SNP begins with the alignment of two different genomes. Despite the clear need for it, the original evidence for the genome itself—the assembly—is not available, and is not linked to the evidence in the Trace Archive. If it were available, many of the polymorphisms already reported could be validated, and many more SNPs might be discovered. Assemblies will also allow centers to better coordinate their gap-closing and finishing efforts, as has been recently noted (Schmutz et al. 2004).

We hope that the availability of the Assembly Archive will encourage human genome sequencers, and sequencers of other genomes, to begin depositing their assemblies into this public resource, where it can be shared by all.

## Abbreviations

SNP: single nucleotide polymorphism
WGS: whole-genome shotgun
